# Concomitant abducens and facial nerve palsies following bone temporal fracture: Case report

**DOI:** 10.1016/j.amsu.2022.104318

**Published:** 2022-08-04

**Authors:** Achraf Amine Sbai, FatimZahra Es-sahli, Azeddine Lachgar, Fahd Elayoubi

**Affiliations:** a Department of Ear Nose and Throat, Mohammed VI University Hospital, Medical School, Mohammed the First University, Oujda, Morocco; b Laboratory of Epidemiology, Clinical Research and Public Health, Faculty of Medicine and Pharmacy of Oujda, Mohammed the First University, Morocco

**Keywords:** Concomitant abducens, Facial nerve palsies, Bone temporal fracture

## Abstract

**Introduction:**

Lesions of the nervus abducens, the 6th cranial nerve tend to be rare, usually occur suddenly following head injuries. The existence of the association of several lesions of the cranial pairs in spite of their rarity must lead the clinician to establish a complete lesion assessment before any cranial trauma

**Case report:**

We describe an illustrative case of sixth nerve palsy associated to facial nerve palsy following a motor vehicle accident. A 36-year-old man had temporal bone fracture after a motor vehicle accident and developed horizontal diplopia and left-sided facial droop, Cranial tomography demonstrated left translabyrinthique bone temporal fracture and fracture of the petrous apex.

**Discussion:**

The petrous apex is an anatomical area rich in vascular and nervous elements. Any damage to this area, whether inflammatory, tumoral or traumatic, as described in this manuscript, can have an irreversible effect if a rapid diagnosis and management is not established.

**Conclusion:**

we report our experience with head trauma with exceptional manifestations, for a better knowledge of these affections, studies with a large number of patients are necessary

## Highlights

• Post traumatic abducens nerve palsy associated with facial paralysis is an exceptional condition.
• Our case report highlights the importance of an early diagnosis and the efficiency of the conservative management.
• The importance of a complete lesion assessment before any cranial trauma despite the rarity of the association of several lesions of the cranial pairs.

## 1 Introduction

The sixth nerve has the longest subarachnoid course of all cranial nerves and innervates the ipsilateral lateral rectus (LR) which abducts the eye. The long span of cranial nerve (CN) VI makes it vulnerable to injury from skull base fractures and increased intracranial pressure [1]. Unilateral abducens nerve palsy forming after head injury occurs at a rate of 1–2.7% and bilateral lesions of the 6th cranial nerve are commonly rare, usually accompanying intracranial or cervical spine injuries [2]. Diplopia (double vision) is the most significant symptom in many patients. It should certainly come to mind in the case of such patients with limited lateral gaze or double vision. In this article, we present a case with a left lesion of the abducens and ipsilateral facial nerve injury developed following a head trauma. Involvement of the 6th and 7th cranial nerves following head injury is seen extremely rarely.

## 2 Case report

### 2.1 Clinical assessment and imaging

A 26-year-old man without any history of medical illness, was referred for horizontal diplopia that worsened on left gaze. He had been admitted for a head trauma caused by a motor vehicle accident the previous day. He had an unremarkable ocular history. He was admitted in the emergency department with a normal Glasgow coma score (GCS = 15). He was alert and oriented, and he demonstrated normal motor and sensory examination of the extremities. And did not demonstrate dysarthria or aphasia. Facial sensation was intact to light touch in V1, V2, and V3 and tongue was midline.

**Fig. 1:**
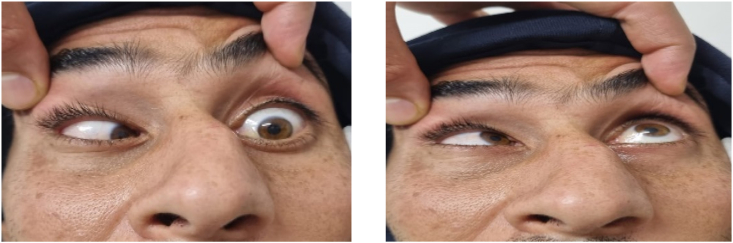
Lack of lateral gaze in left eye attributable to cranial nerve VI injury.

**Fig. 2:**
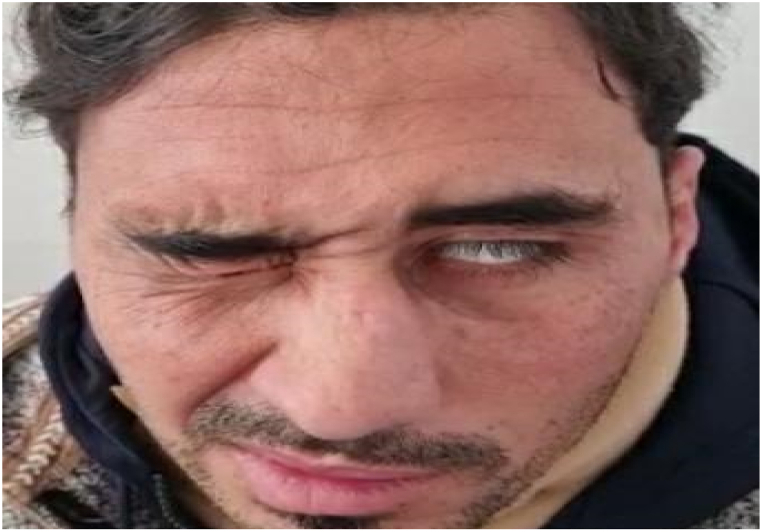
Left facial nerve palsy: Incomplete eye closure, nasolabial fold loss as the result of injury to the facial nerve with barely perceptible mouth and frontal movements.

His ophthalmologic examination revealed a complete CN VI palsy (absent abduction), (Fig. 1), providing the basis for absent lateral gaze with lift eye, he also complained of binocular diplopia on left horizontal gazes, without any reduced visual acuity in both eyes. Left CV VII palsy was noticed and graded as 6/6 House**-**Brackmann (Fig. 2). A pyramid nasal deviation was also obvious (Fig. 2).

There was no hearing loss and the otoscopic examen was normal. Although no evidence of relative afferent pupil defect or CN III palsy was observed. The remainder of the neurologic and physical examination was unremarkable.

**Fig. 3:**
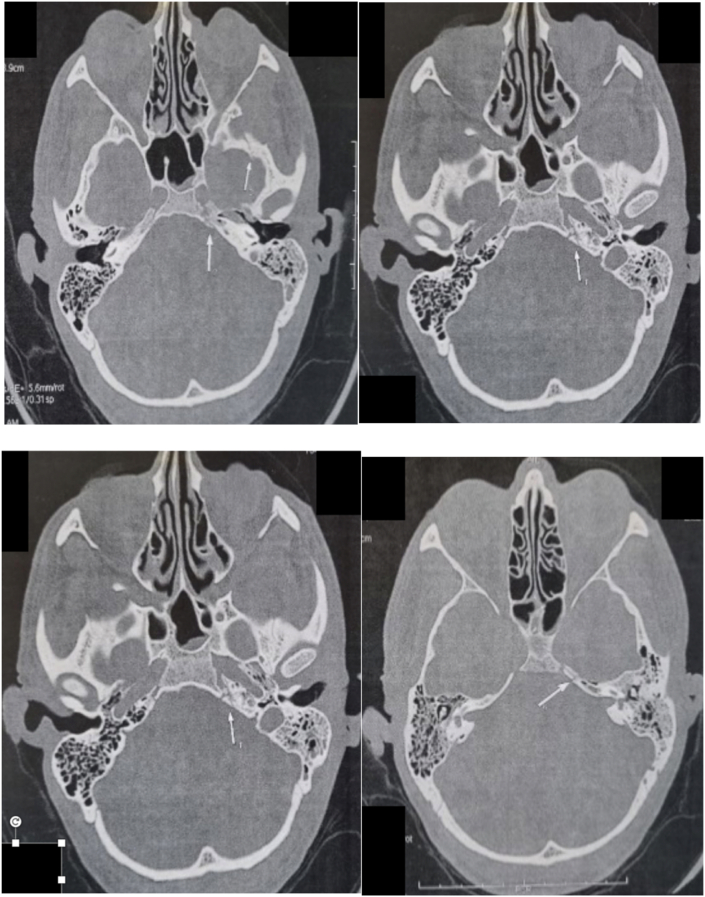
(a and b) Transverse section of cranial CT showed translabyrinthique fracture of the left temporal bone with extension to the left apex petrous.

Brain CT scan showed translabyrinthique fracture of the left temporal bone with extension to the left apex petrous and central skull base including sphenoid lateral wall (Fig. 3).

EMG has demonstrated left PFP with a complete bloc of conduction in the apex petrous.

### 2.2 Management and resolution

Follow-up was coordinated in conjunction with the otolaryngology and ophthalmology services regarding management of CN VI and VII palsies. A conservative management was adopted including corticotherapy, eye protection and facial passive physiotherapy.

The patient recovered facial nerve function to a 2/6 House-Brackmann at 10 weeks’ post-accident, he reported improved ability to abduct the left eye without any residual diplopia, the patient was satisfied with the evolution under medical treatment. Our study has been reported following the SCARE 2020 Checklist criteria [3].

## 3 Discussion

The abducens nerve (cranial nerve VI) is the longest cranial nerve, exits the brainstem at the pontomedullary junction close to the midline and travels anteriorly before turning vertically to ascend in the subarachnoid space over the clivus. It then dissects through the dura until it traverses over the ridge of the petrous bone and under the petroclinoidal (Gruber) ligament. This space is termed Dorello canal and is demarcated by Gruber ligament, the posterior clinoidal process, and the petrous bone. From here, the nerve passes through the cavernous sinus, then the superior orbital fissure to innervate the lateral rectus**.** [4].

The facial (VII) nerve fascicle loops around the nucleus of the sixth (VI) nerve, creating the facial colliculus, and turns anterolaterally to join the eighth nerve in its exit from the lower pons. They then travel upward and laterally to enter the internal auditory meatus**.**

Unilateral abducens nerve palsy is reported to occur in 1–2.7% of patients with head trauma, and is usually isolated [1]. non-isolated cases commonly include bilateral abducens palsy or associated third or fourth nerve palsies; concomitant seventh nerve palsy is relatively rare [2]. Likewise, although traumatic facial nerve palsy is common in the setting of ipsilateral temporal bone fracture, associated abducens palsy has rarely been reported. The reversibility of cranial nerve palsies depends on the type of nerve, the recovery of motor nerves in general is more satisfactory than that of sensorial nerves [5].

Min-Jeong Ji et al. [6] have reported a case of concomitant abducens and facial nerve palsies following a temporal bone fracture, (after a blunt head trauma) the facial nerve palsy has been requiring surgical decompression, while the abducens nerve palsy appeared and recovered without any treatment 2 months later, as same as our case. This spontaneous recovery rate of unilateral traumatic sixth nerve palsy has been estimated at anywhere from 12% to 73% at 6 months [5,7]. Of those patients who will spontaneously recover, the median time to recovery is 90 days. (4) Predictors of no recovery include complete, rather than partial, loss of abduction and bilateral palsy [8].

Management of CN injury is mostly symptomatic. For CN VI injury, occlusion of an eye with an eye pad may be useful to alleviate the symptoms of diplopia. Fresnel prism goggles can realign images in diplopic vision after CN VI injury and aid in rehabilitation. Another therapeutic option is botulinum injection to weaken the medial rectus. If resolution is not observed, strabismus surgery can be considered [9].

The management of traumatic facial nerve injuries is controversial. Cases of complete paralysis in which the onset of paralysis is indeterminate should be treated as immediate in nature. Delayed paralysis or incomplete paresis should be treated medically, with high-dose steroids [10].

A good prognosis should be anticipated in these cases. In the studies by Dahiya et al. and Brodie and Thomson, 100% of patients with delayed onset facial weakness that were selected for conservative treatment recovered function to a House–Brackmann grade II or better [11,12].

Oubhi et al. reported a case of bilateral traumatic facial paralysis with hearing impairment and abducens Palsy after a temporal bone fracture, which was managed conservatively with gratifying results proceeding a medical treatment and early physiotherapy [13].

In our case the patient had a short course of intravenous corticosteroids, with antibiotics Passive facial physiotherapy was started as soon as possible with corneal protection.

Following up was coordinated in conjunction with the otolaryngology and ophthalmology service we noticed a remarkable improvement concerning the left side of the facial palsy, becoming a grade 2 H–B and the abducens palsy had spontaneously regressed after 2 months.

Among the limitations of our case report include that the literature reports only isolated cases, hence the need for studies involving a larger number of cohorts.

The rarity of the association of these 2 lesions makes the literature poor on the subject. Despite the severity of our patient's injury, medical (non-surgical) treatment was successful. This should be taken into account in similar studies in the future to establish codified indications for the management of multiple nerve injuries.

We have started a prospective study allowing the clinical and radiological analysis of the lesions of the cranial pairs in all craniofacial trauma.

## 4 Conclusion

Post traumatic abducens nerve palsy associated with facial paralysis is an exceptional condition. Complete neurologic and ophthalmologic examination in conjunction with analysis of available neuroimaging studies was necessary to arrive at the optimal therapeutic regimen for this patient. Facial nerve recovery is the major concern, as abducens nerve palsy usually recovers spontaneously. This case report highlights the importance of an early diagnosis and the efficiency of the conservative management.

## Sources of funding

This research was not funded.

## Sources of funding

This research was not funded.

## Ethical approval

This is a case report that does not require a formal ethical committee approval. Data were anonymously registered in our database. Access to data was approved by the head of the department.

## Consent

A written informed consent was obtained from the patient for publication of this case report and accompanying images. A copy of the written consent is available for review by the Editor-in-Chief of this journal on request.

## Author contribution

Dr. Achraf Amine SBAI wrote the manuscript.

Dr. FatimZahra ES-SALHI helped in writing and provided surgical data.

Pr. Azeddine LACHKAR helped in writing and literature review.

Pr. Fahd ELAYOUBI helped in writing, supervised the redaction, revised and approved the final draft for publication.

All authors approved the final version of the manuscript.

## Registration of research studies

This is not an interventional study. We only reported the patient's findings from our database as a case report.

## Guarantor

Dr Achraf SBAI.

## Declaration of competing interest

The authors declare no conflicts of interest.
